# Cord Platelet Count of Full-Term Neonates in Relation to ABO Incompatibility and Glucose-6-Phosphate Dehydrogenase Levels: A Retrospective Cohort Study

**DOI:** 10.7759/cureus.30284

**Published:** 2022-10-14

**Authors:** Sameer Y Al-Abdi, Abbas M Al-Omran, Hesham A Shapan

**Affiliations:** 1 Neonatology, King Abdulaziz Hospital, Al-Ahsa, SAU; 2 Neonatology, Almana General Hospital, Al-Ahsa, SAU

**Keywords:** platelet count (plt), abo blood groups, human umbilical cord, glucose-6-phosphate-dehydrogenase deficiency (g6pd), abo incompatibility

## Abstract

Background

The immunoglobulin G of mothers with O blood type may sensitize the platelets of their neonates with A (O-A incompatibility) or B (O-B incompatibility) blood type. As the expression and antigenicity of the B antigen on platelets is less than that of the A antigens, we have hypothesized that platelet count is higher in the O-B incompatibility group compared to the O-A incompatibility group. There is controversy about whether glucose-6-phosphate dehydrogenase (G6PD) deficiency, without evidence of hemolysis, is associated with a lower platelet count than G6PD-normal.

Aim

To assess whether platelet count is higher in the O-B than in the O-A incompatible neonates and whether it correlates with their G6PD levels.

Methods

This study was a retrospective cohort study on a sample of 835 healthy neonates born at ≥38 weeks gestation who were either A or B blood types with mothers that carried the blood type O Rh-positive. The platelet count (thousand per microliter) from umbilical cord venous blood (UCVB) was used. A G6PD level of 11.0 units/gram of hemoglobin (U/g Hb) was considered the lower reference limit. G6PD deficiency was defined as a G6PD level of <3.3 U/g Hb in both sexes. Intermediate G6PD deficiency in females was described as a G6PD level of 3.3-8.8 U/g Hb.

Results

The mean UCVB platelet count was higher in female neonates compared to male neonates (n=389, 283±65 versus n=446, 272±73, p=0.01). The mean UCVB platelet count was higher in the O-B incompatibility group in both male (n=114, 291±82 versus n=103, 266±63) and female neonates (n=83, 303±66 versus n=81, 278±58) with G6PD levels of >8.8 U/g Hb. There was a positive weak correlation between UCVB platelet counts and G6PD levels only in O-B incompatible female neonates (n=176, r=0.23, p=0.002). The partitioning and combined 95% reference intervals (RIs) of the UCVB platelet count were presented.

Conclusion

The platelet count was higher in the O-B incompatibility group compared to the O-A incompatibility group, but only when the G6PD level was >8.8 U/g Hb. A correlation between UCVB platelet count and G6PD levels was found only among O-B incompatible female neonates. These findings may have an important implication in estimating RIs of the UCVB platelet count, however, they need to be confirmed and explored in future research.

## Introduction

Blood type antigens A and B are expressed on the platelets matching the individual's erythrocyte ABO blood type [1-6]. The immunoglobulin G (IgG) of mothers with O blood type may sensitize the platelets of their neonates with A (O-A incompatibility) or B (O-B incompatibility) blood type. Severe neonatal thrombocytopenia due to the mothers' IgG has been reported in four cases [7-10]. As the expression and antigenicity of the B antigen on platelets are less than that of the A antigens [2,6,11], it is plausible to assume that platelet destruction is less in the O-B incompatibility group compared to the O-A incompatibility group. Therefore, we hypothesized that platelet count is higher in the O-B than in the O-A incompatibility group. To our knowledge, this hypothesis has not been studied previously.

Glucose-6-phosphate dehydrogenase (G6PD) is expressed in all human cells. In G6PD-deficient individuals, low G6PD levels have been found in human cells other than erythrocytes, including platelets [12,13]. Erythrocytes and platelet-derived microparticles have been found to be increased in G6PD-deficient individuals [14,15]. These microparticles may cause platelet aggregation, which ultimately causes thrombocytopenia [15,16]. Consequently, the platelet count may be lower in G6PD-deficient individuals compared to G6PD-normal individuals. Some studies have found that G6PD deficiency without evidence of hemolysis is associated with a lower platelet count than G6PD-normal [17-19], but other studies have not found such an association [20,21]. Thus, we conducted this study to assess whether platelet count is higher in the O-B incompatible neonates versus (vs) the O-A incompatible neonates and whether it correlates with their G6PD levels.

## Materials and methods

Routine neonatal care at the study hospital

The routine neonatal care at the study hospital was published in detail previously [22]. Briefly, this care included performing blood grouping and antibody screening for all pregnant women antenatally or during delivery, as well as blood grouping, direct antiglobulin testing, G6PD deficiency screening, and conducting a complete blood cell count (CBC) from umbilical cord venous blood (UCVB) for all neonates. The CBC was performed using CELL-DYN 3700 (Abbott Diagnostics, Santa Clara, CA, USA). If the platelet count from the UCVB sample was <150 109/L, a confirmatory CBC from the peripheral venous blood (PVB) of neonates was performed. 

Study design

This retrospective cohort study was conducted in Almana General Hospital, Al-Ahsa Governorate, Eastern Province, Saudi Arabia. We included a convenience sample of neonates born between 01 January 2013 and 31 December 2021. We included only healthy neonates admitted to the nursery care unit who were born at ≥38 weeks gestation, who underwent UCVB platelet count, G6PD screening, and who had blood type A or B, with mothers who were O Rh-positive. Neonates that had no UCVB CBC (n=60) or no G6PD level measurement (n=19) were excluded from the study. We defined ABO incompatibility as the mother's blood type being O and her neonate's blood type being either A or B, regardless of neonatal direct antiglobulin test results [23-26].

The World Health Organization (WHO) endorses a G6PD level of <30% of the normal as a G6PD deficiency in both sexes and a G6PD level between 30% and 80% of the normal as an intermediate deficiency (heterozygous) in females [27]. For this study, a G6PD level of 11.0 units/gram of hemoglobin (U/g Hb) was considered the lower reference limit [28]. Thus, the G6PD deficiency was defined as a G6PD level of <3.3 U/g Hb in both sexes. The intermediate G6PD deficiency in females was described as a G6PD level of 3.3-8.8 U/g Hb. Medical records of the study neonates were reviewed after obtaining ethical committee approval with a waiver of the consent.

Statistical analysis

The frequency of O-A and O-B incompatibility was reported to be the same among Saudi full-term neonates in the Al-Ahsa Governorate [29]. Thus, a binomial test with a test proportion of 0.5 was run to compare the observed frequency of O-A vs O-B incompatibility and male vs female neonates.

Horn's method for detecting outliers was used [28,30]. Two outliers were detected (639 and 927 thousand per microliter {K/µL}), which were replaced by their confirmatory PVBs (224 and 248 K/µL, respectively). There were 19 UCVB platelet count measurements <150 (101-149) K/µL. Out of these, nine had their PVB platelet count <150 K/µL, eight >150 K/µL, and two had no confirmatory PVB CBC measurements. We replaced the 17 UCVB platelet measurements of <150 K/µL with their confirmatory PVB platelet count. The timing of PVB was, on average, within the first eight hours of life.

The UCVB platelet counts and G6PD levels were not normally distributed. Consequently, an ultra-fine transformation was performed to normalize the UCVB platelet count and G6PD level distributions [31]. A Student’s t-test was used and data were presented as the mean ± standard deviation (SD). Pearson's correlation was run to examine the relationship between UCVB platelet count and G6PD levels. A 1000‐iteration bootstrap was used to calculate Pearson’s correlation coefficient's 95% confidence interval (CI). A correlation of <0.30 was considered weak [32]. A Chi-square test and Fisher’s exact test were used to analyze categorical variables, when appropriate. The 95% reference interval (RI) of the UCVB platelet count was estimated according to the International Federation of Clinical Chemistry and the Clinical and Laboratory Standards Institute (CLSI) for reference interval estimation [33,34]. A 500‐iteration bootstrap non-parametric method was used to estimate the 95% RIs and their 90% CIs [35]. We considered a two-tailed p-value of <0.05 as statistically significant for all the tests. The analysis was performed using IBM SPSS Statistics, version 20.0 (IBM Corp., Armonk, NY) and RefVal 4.11 [35].

## Results

A total of 835 neonates met our inclusion and exclusion criteria. Although there was a trend of fewer females than males in the cohort (389 {46.6%} vs 446 {53.4%}), the difference was insignificant (binomial p-value = 0.053). The frequency of O-A and O-B incompatibility was the same (428 {51.3%} vs 407 {48.7%}, binomial p-value = 0.51). Table 1 depicts the baseline characteristics and laboratory findings of the O-A and O-B incompatible neonates. Among neonates with a G6PD level of <3.3 U/g Hb, male neonates were more prevalent than female neonates (100 {22.4%} vs 53 {13.6%}, p = 0.001). Contrarily, among neonates with a G6PD level of 3.3-8.8 U/g Hb, the rate of females was higher than male neonates (172 {44.2%} vs 129 {28.9%}, p <0.001). The female neonates with a G6PD level of 3.3-8.8 U/g Hb were observed more in the O-A than O-B incompatibility group (Table 1). The rate of male and female neonates was the same among those with a G6PD level of >8.8 U/g Hb (217 {48.7%} vs 164 {42.2%}, p = 0.06).

**Table 1:** Comparison of baseline characteristics and laboratory findings between neonates with O–A and O–B incompatibility. Data are presented as number (%) unless specified otherwise. G6PD (Glucose-6-phosphate dehydrogenase). U/g Hb (Units/gram of Hemoglobin).

Stratum	O–A (n=428)	O­–B (n=407)	P-Value
38 weeks' gestation	116 (27.1%)	119 (29.2%)	0.49
39 weeks' gestation	140 (32.7%)	159 (39.1%)	0.06
40 weeks' gestation	131 (30.6%)	107 (26.3%)	0.17
41 weeks' gestation	38 (8.9%)	21 (5.2%)	0.04
42 weeks' gestation	3 (0.7%)	1 (0.2%)	0.34
Birth weight (mean±SD), grams	3222±386	3189±399	0.22
Female neonates	213 (49.8%)	176 (43.2%)	0.06
Spontaneous vaginal delivery	321 (75.0%)	317 (77.9%)	0.33
Assisted vaginal delivery	11 (2.6%)	13 (3.2%)	0.59
Elective Cesarean delivery	28 (6.5%)	26 (6.4%)	0.93
Emergency Caesarean delivery	68 (15.9%)	51 (12.5%)	0.17
Males and females with G6PD level > 8.8 U/g Hb	184 (43.0%)	197 (48.4%)	0.12
Males with G6PD level 3.3–8.8 U/g Hb	64 (15.0%)	65 (16.0%)	0.71
Females with G6PD level 3.3–8.8 U/g Hb	104 (24.3%)	68 (16.7%)	0.01
Males and females with G6PD level < 3.3 U/g Hb	76 (17.7%)	77 (18.9%)	0.66

The mean UCVB platelet count was higher in female than male neonates (283±65 K/µL vs 272±73 K/µL, p = 0.01). The mean UCVB platelet count of O-A incompatible female neonates was higher than that of their counterpart male neonates (n = 213, 279±64 K/µL vs n = 215, 266±67 K/µL, p = 0.03). The mean UCVB platelet count was the same in the O-B incompatible female and male neonates (n = 176, 288±66 K/µL vs n = 231, 279±78 K/µL, p = 0.09). In neonates with a G6PD level of >8.8 U/g Hb, the mean UCVB platelet count was higher in the O-B incompatible neonates in both sexes (Tables 2-3).

**Table 2:** Comparisons between umbilical cord platelet count in 446 O–A and O–B incompatible male neonates. G6PD (Glucose-6-phosphate dehydrogenase). U/g Hb (Units/gram of Hemoglobin). ^a ^Comparison between these two means was statistically significant with p = 0.02.

Stratum	Total (%)	O–A (n=215)		O­–B (n=231)		P-Value
		number	mean±SD	number	mean±SD	
G6PD level > 8.8 U/g Hb	217 (48.7%)	103	266±63	114	291±82^a^	0.02
G6PD level 3.3–8.8 U/g Hb	129 (28.9%	64	265±69	65	262±62^a^	0.61
G6PD level < 3.3 U/g Hb	100 (22.4%)	48	266±73	52	272±84	0.79

**Table 3:** Comparisons between umbilical cord platelet count in 389 O–A and O–B incompatible female neonates. G6PD (Glucose-6-phosphate dehydrogenase). U/g Hb (Units/gram of Hemoglobin). ^a^ Comparison between these two means was statistically significant with p = 0.04. ^b^ Comparison between these two means was statistically significant with p = 0.002.

Stratum	Total (%)	O–A (n=213)		O­–B (n=176)		P-Value
		number	mean±SD	number	mean±SD	
G6PD level > 8.8 U/g Hb	164 (42.2%)	81	278±58	83	303±66^a,b^	0.02
G6PD level 3.3–8.8 U/g Hb	172 (44.2%)	104	280±62	68	280±64^a^	>0.99
G6PD level < 3.3 U/g Hb	53 (13.6%)	28	280±84	25	257±59^b^	0.34

Tables 2-3 depict the means of the UCVB platelet counts and show that the O-A incompatible neonates were the same across all the G6PD level strata in both sexes (all p values >0.05). There was no correlation between UCVB platelet count and G6PD levels among O-A incompatible male or female neonates (p values >0.05). The mean UCVB platelet count of O-B incompatible male neonates with a G6PD level of >8.8 U/g Hb was higher than their counterparts with a G6PD level of 3.3-8.8 U/g Hb (Table 2). Among O-B incompatible male neonates, there was no correlation between UCVB platelet counts and G6PD levels (n = 231, r = 0.12, 95% CI: -0.03 to 0.26, p = 0.07).

As shown in Table 3, the mean UCVB platelet count of the O-B incompatible female neonates with a G6PD level of >8.8 U/g Hb was higher than their counterparts with a G6PD level of ≤8.8 U/g Hb. There was a positive weak correlation between UCVB platelet counts of O-B incompatible females and their G6PD levels (n = 176, r = 0.23, 95% CI: 0.06 to 0.37, p = 0.002).

Table 4 depicts partitioning and combined 95% RIs of the UCVB platelet counts among neonates with a G6PD level of >8.8 U/g Hb. The lower reference limit (2.5th percentile) of sex partitioning RIs was higher in females, whereas the upper reference limit (97.5th percentile) was higher in male neonates. Among the ABO incompatibility partitioning RIs, the lower reference limit was almost the same in the O-A and O-B incompatible neonates, but the upper reference limit was higher in the O-B than in O-A incompatible neonates.

**Table 4:** Partitioning and combined 95% reference interval (RI) for platelet count (thousand per microliter) measured from umbilical cord venous blood of neonates with G6PD level >8.8 U/g Hb. G6PD (Glucose-6-phosphate dehydrogenase). U/g Hb (units/gram of hemoglobin).

Stratum	95% Reference Interval in thousand per microliter		Number of values (%) outside combined RI
	2.5^th^ percentile (90% confidence interval)	97.5^th^ percentile (90% confidence interval)	
Male neonates (n=217)	156 (151–172)	443 (420­–526)	31 (14.3%)
Female neonates (n= 164)	175 (151–193)	420 (393–491)	Zero
O–A incompatible neonates (n=184)	159 (153–176)	387 (376–425)	Zero
O–B incompatible neonates (n=197)	166 (151–178)	490 (428–549)	13 (6.6%)
Combined RI (n=381)	160 (154–175)	429 (416–465)	Not applicable

## Discussion

The present study confirmed our hypothesis that the platelet count was higher in the O-B incompatibility group compared to the O-A incompatibility group, but only when the G6PD level was higher than 8.8 U/g Hb. It demonstrated that the correlation between UCVB platelet count and G6PD levels was only among O-B incompatible female neonates. These findings may have an important implication in estimating RIs of the UCVB platelet count.

The UCVB platelet count of O-A incompatible male and female neonates was constant among different G6PD strata. However, the UCVB platelet count of O-B incompatible male and female neonates increased as G6PD levels increased, except for male neonates with G6PD levels between 3.3-8.8 U/g Hb. Thus, the correlation between the UCVB platelet count and G6PD levels was significant only among O-B incompatible female neonates. Unfortunately, we have no plausible biological explanation for why the relationship between UCVB platelet count and G6PD level was different in O-A vs O-B incompatibility groups. We also have no reason why the UCVB platelet of O-B incompatible male neonates with G6PD levels between 3.3-8.8 U/g Hb was lower than their counterparts with a G6PD level of <3.3 U/g Hb. Nevertheless, further research is required to explore the biological plausibility of our findings, which have not been elucidated because of the lack of essential explanatory data.

We are unaware of previous neonatal or pediatric research on the relationship between platelet count and ABO blood types. A very large Italian adult study showed that platelet count was significantly lower in the B blood type than in other ABO blood types [36]. However, comparing and contrasting our study with the Italian study is difficult.

The UCVB has been promoted as a wise choice for blood investigation in neonates, including platelet count [37-39]. For that reason, 95% RIs of UCVB platelet count have started appearing in the literature recently [40,41]. We, as well as a Turkish (mean, 256.1±58.9 vs 244.5±55.9 K/µL) and an Ethiopian study (median, 241.5 vs 230.0 K/µL), found that the UCVB platelet count was higher in female neonates compared to male neonates [40,41]. The Turkish study estimated the UCVB platelet count’s 95% RIs as 129-353 K/µL in 974 male neonates and 128-367 K/µL in 924 female neonates [40]. The Ethiopian study estimated the UCVB platelet count’s 95% RI among 67 male neonates as 146.7-466.2 K/µL and 146.0-418.2 K/µL among 72 female neonates [41]. Our estimated 95% RI for male (156-443 K/µL) and female (175-420 K/µL) neonates differed from that estimated in both studies, particularly the Turkish study. These differences could be due to pre-analytical reasons, including sample collection and processing, analytical method, and instrumentation. We used CELL-DYN 3700 (Abbott Diagnostics, Santa Clara, CA, USA) for CBC analysis. In contrast, the Turkish study used Beckman Coulter AcT diff2 (Brea, CA, USA) and the Ethiopian used Sysmex KX-21 N (Sysmex Corporation, Kobe, Japan) for CBC analysis. Thus, the CLSI endorses that if someone wishes to adopt a RI estimated by others, then the analytical system and the test subject population should be comparable in both settings [34].

A partitioning (subgroups) RI is recommended to be used instead of a combined (common) RI if any of the two values at the lower and upper end of the distributions outside the combined RI are ≥4.1% [42,43]. We observed that 14.3% of the UCVB platelet count measurements in the 95% RIs of sex partitioning and 6.6% in the 95% RIs of ABO incompatibility partitioning were outside the combined 95% RI. Therefore, these observations suggest that the partitioning of 95% RI, particularly the sex partitioning, should be used instead of the combined 95% RI in our population. 

The G6PD-Mediterranean (WHO class II, <10% of normal activity) comprises 84%, and the G6PD-A- (WHO class III, 10-60% of normal activity) comprises 5.8% of G6PD mutations in the Al-Ahsa Governorate [44]. Albagshi et al. estimated the prevalence of G6PD deficiency to be 26% in males and 9.9% in female neonates in the Al-Ahsa Governorate based on a qualitative fluorescent spot test (FST) [45]. As the cuff-off value of the FST is 2.1 U/g Hb [46], our observed rate of G6PD level <3.3 U/g Hb in male (22.4%) and in female (13.6%) neonates was comparable to that reported by Albagshi et al.

In addition to being a retrospective study, several limitations of the present study should be noted. First, we had no data on the timing of umbilical cord sampling. It has been shown that the platelet count was slightly higher if the blood sampling was performed after 10 minutes of placental delivery from the umbilical vein near or on the placental surface or after 30 minutes from an umbilical cord segment [47]. Second, although our total sample size was large, it was not large enough in certain strata. As the correlation coefficient was 0.12 for the correlation between UCVB platelet counts and G6PD levels among the 231 O-B incompatible male neonates, a minimum sample size of 400 would be needed for the two-tailed 0.05 statistical significance [48]. Third, we diagnosed intermediate (heterozygous) G6PD deficient female neonates by a qualitative assay, whereas the gold standard diagnostic tests are genotyping and cytochemical staining for intracellular G6PD activity [49]. Thus, we are unsure if our observed rate of 44.2% of female neonates with G6PD levels between 3.3-8.8 U/g Hb is true. Unfortunately, there were no previously published data from our Governorate that we could compare our rate to. An alternative way of checking our observed rate is to compare it with an expected rate. The Hardy-Weinberg formula, which assumes no consanguinity, can be used to calculate the expected rate [50]. However, since consanguinity is common in our population [51], the Hardy-Weinberg formula cannot be used to calculate in our setting. Fourth, we did not adjust p-values for the multiple comparisons, which might increase the risk of false statistical significance, particularly for the p-values <0.05.

## Conclusions

The present study confirmed our hypothesis that the platelet count was higher in the O-B incompatibility group compared to the O-A incompatibility group, but only when the G6PD level was higher than 8.8 U/g Hb. Our results demonstrated a correlation between UCVB platelet count and G6PD levels only among O-B incompatible female neonates. The difference between UCVB platelet count in O-A vs O-B incompatibility groups appeared to be G6PD level-dependent. The relationship between UCVB platelet counts and G6PD levels may depend on sex and ABO incompatibility. These findings may have an important implication in estimating RIs of the UCVB platelet count. Unfortunately, we have no biological explanations for these findings. Thus, these findings need to be confirmed and explored in future explanatory research.
